# Using Object Detection Technology to Identify Defects in Clothing for Blind People

**DOI:** 10.3390/s23094381

**Published:** 2023-04-28

**Authors:** Daniel Rocha, Leandro Pinto, José Machado, Filomena Soares, Vítor Carvalho

**Affiliations:** 1 Algoritmi Research Centre/LASI, University of Minho, 4800-058 Guimarães, Portugal; id8057@alunos.uminho.pt (D.R.); fsoares@dei.uminho.pt (F.S.); 2 2Ai, School of Technology, Polytechnic Institute of Cávado and Ave, 4750-810 Barcelos, Portugal; a16207@alunos.ipca.pt; 3 INL—International Nanotechnology Laboratory, 4715-330 Braga, Portugal; 4 MEtRICs Research Centre, University of Minho, 4800-058 Guimarães, Portugal

**Keywords:** blind people, clothing defect detection, object detection, deep learning, YOLOv5

## Abstract

Blind people often encounter challenges in managing their clothing, specifically in identifying defects such as stains or holes. With the progress of the computer vision field, it is crucial to minimize these limitations as much as possible to assist blind people with selecting appropriate clothing. Therefore, the objective of this paper is to use object detection technology to categorize and detect stains on garments. The defect detection system proposed in this study relies on the You Only Look Once (YOLO) architecture, which is a single-stage object detector that is well-suited for automated inspection tasks. The authors collected a dataset of clothing with defects and used it to train and evaluate the proposed system. The methodology used for the optimization of the defect detection system was based on three main components: (i) increasing the dataset with new defects, illumination conditions, and backgrounds, (ii) introducing data augmentation, and (iii) introducing defect classification. The authors compared and evaluated three different YOLOv5 models. The results of this study demonstrate that the proposed approach is effective and suitable for different challenging defect detection conditions, showing high average precision (AP) values, and paving the way for a mobile application to be accessible for the blind community.

## 1. Introduction

Visual impairment, e.g., blindness, can have a significant impact on the psychological and cognitive functioning of an individual. Several studies have shown that vision impairment is associated with a variety of negative health outcomes and a poor quality of life [1,2]. Additionally, blindness currently affects a significant number of individuals, and thus it should not be assumed as a minor concern for society. According to a recent study, there are 33.6 million people worldwide suffering from blindness, which clearly shows the dimension of this population group [3].

The use of assistive technology can help in mitigating the negative effects of blindness and improve the quality of life of people who are blind. Although there has been a proliferation of smart devices and advancements in cutting-edge technology for blind people, most research efforts have been directed towards navigation, mobility, and object recognition, leaving aesthetics aside [4,5,6]. The selection of clothing and preferred style for different occasions is a fundamental aspect of one’s personal identity [7]. This has a significant impact on the way we perceive ourselves, and on the way we are perceived by others [7,8]. Nonetheless, individuals who are blind may experience insecurity and stress when it comes to dressing-up due to a lack of ability to recognize the garments’ condition. This inability to perceive visual cues can make dressing-up a daily challenge. In addition, blind people may have a higher probability of clothing staining and tearing due to the inherent difficulties in handling objects and performing daily tasks. In particular, detecting stains in a timely manner is crucial to prevent them from becoming permanent or hard to remove. Despite the promising potential of technological solutions in the future, significant challenges still need to be overcome. The lack of vision makes it challenging for these individuals to identify small irregularities or stains in the textures or fabrics of clothing, and they rely on others for assistance. This was the fundamental baseline behind the motivation for this work; namely, how to enable blind people to feel equally confident with what they wear, without permanently needing help. This perception is still missing in blind people’s lives, and clothing still represents a daily challenge.

Hence, this study addresses the challenge of identifying defects in clothing, i.e., holes and stains, using computer vision advances, namely object detection techniques, contributing to a higher standard of living for blind people when deciding what to wear. Deep learning methods have become a powerful approach for directly acquiring feature representations from data, resulting in significant advancements in the domain of object detection [9,10]. The proposed methodology is an extension of a previous work by the authors [11,12,13,14,15,16], and makes notable contributions to the field by offering: (i) a compilation of techniques utilized in object detection for the identification of clothing defects, (ii) an annotated dataset of clothing defects that can be utilized by the research community for additional studies, and (iii) a comparison of different versions of YOLOv5 networks for the detection of defects on clothing. The validation of the developed work was conducted through a collaboration with the ACAPO and the Association of Support for the Visually Impaired of Braga (AADVDB), which helped to identify key areas for improvement. The findings of this research envisage future practical applications to complement the presented algorithms and methodology, namely a mobile application and a mechatronic system, i.e., an automatic wardrobe. The following sections include an overview of related work (Section 2), the methodology (Section 3), results and discussion (Section 4), and conclusions and future work (Section 5). 

## 2. Related Work

Defect detection in clothing remains a barely addressed topic on the literature. However, if the scope of the topic is expanded to the industry, some interesting works have been carried out, mainly regarding the fabric quality control in the textile industry. Such quality control approach still plays an important role in the industry, and can be an appealing starting point for defect detection in clothing with other purposes in sight [17]. 

Based on the aforementioned premise, a quick literature survey allows perceiving that machine vision based on image processing technology has replaced manual inspection, and allows for reducing costs and increasing the detection accuracy. An integral part of modern textile manufacturing is the automatic detection of fabric defects [18]. More recently, due to their success in a variety of applications, deep learning methods have been applied to the detection of fabric defects [19]. A wide range of applications were developed using convolutional neural networks (CNNs), such as image classification, object detection, and image segmentation [20]. Defect detection using convolutional neural networks can be applied to several different objects [21,22,23]. Comparatively to traditional image processing methods, CNNs can automatically extract useful features from data without requiring complex feature designs to be handcrafted [24]. Zhang et al. [25] presented a comparative study between different networks of YOLOv2, with proper optimization, in a collected yam-dyed fabric defect dataset, achieving an intersection over union (IoU) of 0.667. Another method, unsupervised, based on multi-scale convolutional denoising autoencoder networks, was presented by Mei et al. [26]. A particularity of this approach is the possibility of being trained with only a small number of defects, without label ground truth or human intervention. A maximum accuracy of 85.2% was reported from four datasets. A deep-fusion fabric defect detection algorithm, i.e., DenseNet121-SSD (Densely Connected Convolutional Networks 121-Single-Shot Multi-Box Detector), was proposed by He et al. [27]. By using a deep-fusion method, the detection is more accurate, and the detection speed becomes more efficient, achieving a mean average precision (mAP) of 78.6%. 

Later, Jing et al. [28] proposed a deep learning segmentation model, i.e., Mobile-Unet, for fabric defect segmentation. Here, a benchmark is performed with conventional networks on two fabric image databases, the Yarn-dyed Fabric Images (YFI) and the Fabric Images (FI), allowing to reach IoU values of 0.92 and 0.70 for YFI and FI, respectively. A novel model of a defect detection system using artificial defect data, based on stacked convolutional autoencoders, was then proposed by Han et al. [29]. Their method was evaluated through a comparative study with U-Net with real defect data, and it was concluded that actual defects were detected using only non-defect and artificial data. Additionally, an optimized version of the Levenberg–Marquardt (LM)-based artificial neural network (ANN) was developed by Mohammed et al. for leather surfaces [30]. The latter enables the classification and identification of defects in computer vision-based automated systems with an accuracy of 97.85%, compared with 60–70% obtained through manual inspection. Likewise, Xie et al. [31] proposed a robust fabric defect detection method, based on the improved RefineDet. Three databases were used to evaluate their study. Additionally, a segmentation network with a decision network was proposed by Huang et al. [32], with the reduced number of images needed to achieve accurate segmentation results being a major advantage. Furthermore, a deep learning model to classify fabric defects in seven categories based on CapsNet was proposed by Kahraman et al. [33], achieving an accuracy of 98.71%. 

Table 1 summarizes the main results of the aforementioned works, including the datasets used.

The results presented in Table 1 demonstrate a lack of standardization in the evaluation metrics and datasets between studies, leading to difficulties in accurately comparing results. This can be attributed to the diversity of tasks in defect detection, including defect classification, defect location, defect segmentation, and defect semantic segmentation, each requiring distinct metrics for evaluation. Furthermore, the studies are focused on one-stage and two-stage detectors, without a comparative study between them. One-stage detectors, such as You Only Look Once (YOLO) [34] and the Single-Shot Detector (SSD) [35], are known for their speed, but also for their lower accuracy compared to two-stage detectors, such as Faster R-CNN [36] and Mask R-CNN (region-based convolutional neural network) [37]. Two-stage detectors offer improved accuracy, but at the cost of a slower performance. 

**Figure 1:**
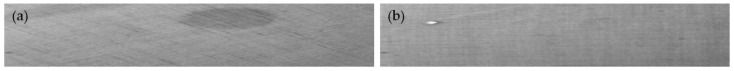
Examples of defects from the TILDA dataset: (**a**) large oil stain in the upper right corner, and (**b**) medium-sized hole in the upper left corner.

Despite the similarities between clothing and textiles, a new approach is needed for detecting defects in clothing, especially to assist blind people. For that, different types of images must be analyzed, other than just textiles, resulting in the creation of new datasets. In the textile industry, fabrics usually emerge from the manufacturing process in a roll and undergo stretching, augmenting the detection of defects. Furthermore, the magnification of images to fit the fabrics coming off the roll can greatly amplify any defects present, as depicted in Figure 1.

**Figure 2:**
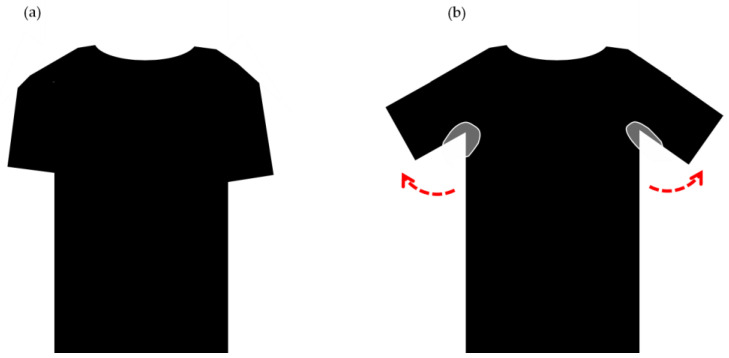
Visibility of the defects on clothing: (**a**) imperceptible sweat stain and (**b**) visible sweat stain.

It becomes clear that a comprehensive dataset that captures the entirety of a garment can provide crucial insights into identifying defects in the piece as a whole, thus, leading to significant advancements in this field. Furthermore, textile fabrics’ datasets may not capture important clothing features, such as wrinkles, patterns, and buttonholes, which can present a significant challenge during the analysis, since defects can be hidden in the wrinkles of the clothes, or simply hidden by the way the garment was folded or stored, as illustrated in Figure 2. 

This means that each clothing piece can be interpreted as a different object, since its shape and color can significantly vary. Such particularity does not occur with rigid objects and objects whose color does not change upon use. At present, and to the best knowledge of the authors, the literature still lacks a system that can automatically identify defects in clothing, an essential support tool for blind individuals to efficiently manage their wardrobe on a daily basis. Aiming at addressing this issue, a solution that utilizes a one-stage detector (YOLOv5) [38] was fine-tuned specifically for this purpose, in line with other research studies that have also efficiently employed YOLOv5 in their research [39,40]. Object detection was chosen over semantic segmentation because the presence of the defect does not require identifying details such as color, origin, type, diameter/area, or any other information that requires labeling every pixel in the image. This means that blind people only need to be informed about the presence of the imperfection, rather than the intricate details of the image. By simplifying the problem to object detection, the solution provides a practical and efficient way for blind people to independently manage their appearance and do so with confidence. Moreover, the proposed solution demonstrates that computer vision can be employed to analyze and overcome this challenge, while opening the door to the possibility of becoming accessible to the blind community through a mobile application.

## 3. Methodology

**Figure 3:**
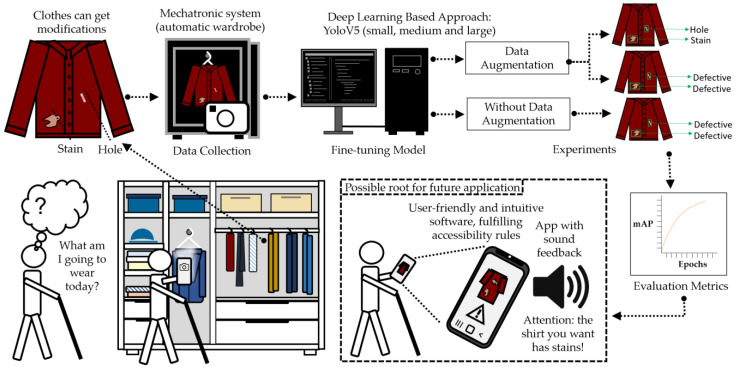
Overview of the proposed methodology for defect detection on clothing, including image acquisition, data collection, model tuning, experiments, evaluation metrics, and future application.

The methodology used for the development of the defect detection system was based on three main components: (i) the increase of the data collection based on the previous work [16], (ii) the introduction of data augmentation, and (iii) the fine-tuning network YOLOv5 architecture, ensuring a possible route for a future automatic application (see Figure 3).

Further details on the data collection procedure, optimized algorithms, and evaluation metrics adopted for solving this task are presented in the following sections.

### 3.1. Data Collection

To the best knowledge of the authors, the previous research on clothing category classification and stain detection [16] remains the only dataset that specifically focus on defects in clothing. Consequently, it was found necessary to expand the existing dataset and evaluate novel neural networks to improve the previous work. Individuals’ clothing collections were the source of the data, which was then deliberately altered through the creation of defects, and manually labeled. The dataset was enhanced by adding stain defects and incorporating hole defects, resulting in ca. 340 images. As a result, each individual garment may exhibit several defects, distributed throughout diverse regions of the attire, namely on the backside, thereby resulting in an aggregate quantity of ca. 647 defects. Table 2 shows the representation of each defect class. 

**Figure 4:**
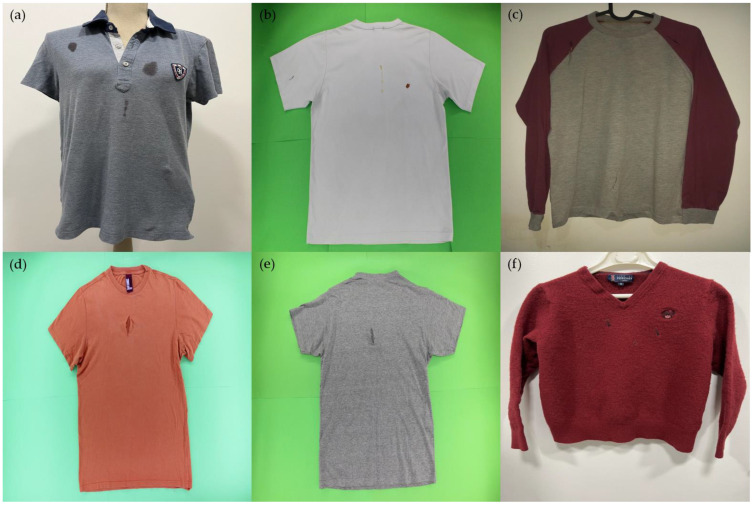
Examples of sample images from the dataset, demonstrating different types of defects and distributions, with variations in backgrounds and illumination conditions: (**a**) multiple stain defects, (**b**) a combination of stain and hole defects, (**c**) multiple hole defects, (**d**) a single hole defect, (**e**) hole defect on the backside, and (**f**) multiple hole defects.

Moreover, despite the authors’ intention of capturing the clothing items in a controlled setup, i.e., all images taken by blind people are placed in an automatic wardrobe [41] with one item of clothing at a time, including garment rotation, illumination, and multiple capture perspectives, various backgrounds and capturing perspectives were used to ensure that future images would meet those conditions. Representative images of the dataset are depicted in Figure 4. 

### 3.2. Data Augmentation

**Figure 5:**
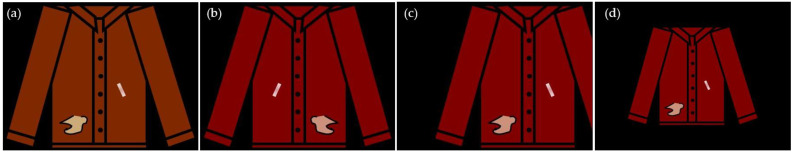
Data augmentation features in training: (**a**) hue-saturation-value (HSV), (**b**) horizontal flipping, (**c**) translation, and (**d**) scaling.

The small scale of the defined dataset was found to be a challenge for future applications. To overcome this limitation and enable the model to generalize from various perspectives, data augmentation was employed to expand the dataset size. Through the augmentation process, a range of transformations, such as horizontal flipping, scaling, translation, and hue-saturation-value (HSV) changes, were applied to the images (Figure 5). The primary goal of these transformations was to replicate real-world contextual scenarios that often go unnoticed by individuals with visual impairments, such as changes in lighting, color, and orientation, during inspection. This strategy resulted in the creation of novel and diverse images based on the original dataset. 

### 3.3. Deep Learning-Based Approach

The deep learning-based object detection technique was used to detect defects in clothing images. This method involves taking an image as input and creating a bounding box that indicates the defect’s location. To accomplish this, a deep learning framework for object detection was utilized via transfer learning, specifically the fine-tuning, i.e., reusing a model that was initially developed for a specific situation and using it as a starting point for another model, aiming at addressing a different problem [42]. In the field of deep learning, transfer learning is widely used due to the significant resources and time required for training neural networks. By leveraging pre-trained models, transfer learning optimizes the performance when training the second model.

In this study, the YOLOv5—specifically, the small, medium, and large models with different layer depths, real-time performance, and detection accuracy—was utilized to assess the difficulties involved in detecting defects using novel data. The YOLOv5 object detection algorithm represents a continuous refinement and enhancement of the YOLO series [34,43,44], where the accuracy of detection has shown noteworthy improvement and, in certain instances, outperforms two-stage detectors. Despite detection accuracy being less of a priority, YOLOs are widely adopted in various applications due to their faster inference speed [45].

To evaluate the challenges of proposing an automatic algorithm for detecting and categorizing clothing defects, three different experiments were conducted: (1) detection of defects on clothing, (2) detection of defects on clothing using data augmentation, and (3) detection and classification of the defects using data augmentation. 

### 3.4. Evaluation Metrics

The proposed methodology was evaluated using standard metrics for object detection competitions, such as MicroSoft Common Objects in Context (MSCOCO) [46] and Pascal Visual Object Classes (PASCAL VOC) [47] challenges, which include average precision (AP) and mean average precision (mAP). Since this methodology is intended for practical applications, where only the presence of defects is important, regardless of their exact location, these metrics were computed using an IoU threshold of 0.50, as shown in Equation (1):

(1) $$IoU=\frac{Area\;of\;Overlap}{Area\;of\;Union}$$

Equations (2) and (3) can be utilized to compute precision and recall using the preceding IoU. Specifically, precision (P) can be obtained by calculating the proportion of accurately predicted positive observations to the total predicted positive observations:

(2) $$P=\frac{TP}{TP+FP}$$

Furthermore, the recall (R) can be determined as the ratio of accurately predicted positive observations to the number of observations present in the actual class:

(3) $$R=\frac{TP}{TP+FN}$$

The TP, FP, and FN indicate the number of true positives (TP), false positives (FP), and false negatives (FN), respectively. Ultimately, a precision–recall curve (PR) was produced for the object class, and the area under the curve indicated the model’s average precision (AP).

## 4. Results and Discussion

In this section, a quantitative assessment of the proposed approach is conducted. Specifically, the experiments mentioned in Section 3.3 are examined in detail in the following sections.

To carry out the evaluation, the dataset was divided into three distinct groups: one for training, another for validation, and a third for testing. These groups were split in a ratio of 70%, 20%, and 10%, respectively. By doing so, it can be determined whether the network can effectively generalize to unseen data and be used for defect detection in clothing. All reported results are based on the best generalization achieved in the experiments. The experiments were set to run for 400 epochs, a range sufficient for convergence, and combined with the early stopping technique, which halts the training process if no improvement is observed for 10 epochs, effectively preventing overfitting. The tests were carried out on a server featuring an Intel(R) Xeon(R) Gold 6140 CPU 2.30 GHz processor, 128 GB of RAM, and a NVIDIA Tesla V100-PCIE-16 GB computing GPU. Table 3 presents the uniform hyper-parameters used throughout the training process in order to facilitate a comprehensive comparison of the different networks.

### 4.1. Clothing Defect Detection

The first experiment consisted of performing the fine-tuning of the models using the gathered dataset, with the specific goal of detecting defects on clothing. Table 4 shows the resulting outcomes.

According to Table 4, YOLOv5l6 had a superior AP at IoU = 0.50 (0.73) when compared to the other models. This outcome may be attributed to the number of undetected defects, as indicated by the recall (0.60) derived from Equation (3). In other words, the model’s predictions contained false negatives, which adversely impacted the AP. On the other hand, the high precision (0.86) indicates that the model had fewer instances of false positives. However, YOLOv5s6, despite its high precision, had a lower recall value (0.41), which negatively affected the average precision. Although the medium model’s generalization (YOLOv5m6) was superior to that of the small model (YOLOv5s6), its average precision was still influenced by the recall. 

### 4.2. Clothing Defect Detection with Data Augmentation

The second stage of the development of the defect detection system aimed at enhancing the performance of the model through data augmentation. Table 5 presents the main outcomes of the second experiment based on the introduction of data augmentation.

The results of Table 5 reveal a noteworthy improvement in the models’ generalization as a result of data augmentation. Specifically, the YOLOv5m6 model exhibited the most significant improvement, with an 8% increase in AP to 0.74, compared to the previous experiment. This improvement is a notable finding, indicating that data augmentation had a substantial positive impact on the generalization performance of the model. This can be primarily attributed to the reduced number of false negative predictions, as evidenced by the recall values. 

**Figure 6:**
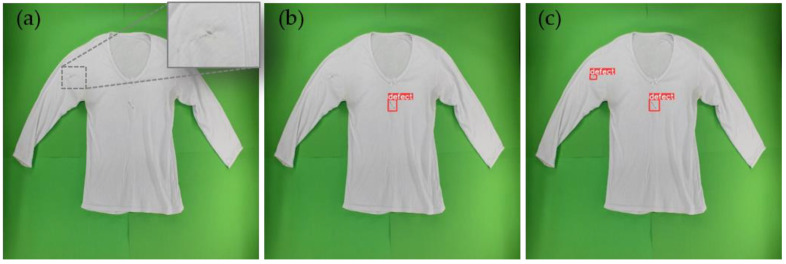
Example of a defect undetected by the YOLOv5m6 model that was subsequently identified with the aid of data augmentation: (**a**) original image, (**b**) predicted image from model YOLOv5m6 without augmentation, and (**c**) predicted image from model YOLOv5m6 with augmentation.

Figure 6 illustrates an example of a defect that went undetected by the YOLOv5m6 model but was subsequently identified with the aid of data augmentation.

### 4.3. Clothing Defect Detection and Classification with Data Augmentation

The third component of the development of the defect detection system included defect classification, enabling the evaluation of the type of defect. Table 6 presents the main performance results for each model, including the average precision (AP) for each class, i.e., holes and stains, as well as the mean average precision for both classes.

**Figure 7:**
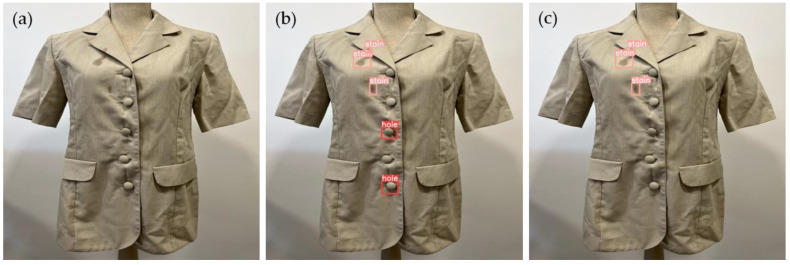
Example of a misinterpretation of a defect: (**a**) original image, (**b**) predicted image from model YOLOv5m6, and (**c**) predicted image from model YOLOv5l6.

The performance results from Table 6 indicate that stains were more accurately detected than holes, considering all metrics, which might be attributed to the inherent difficulty in detecting holes in clothes, even to the human eye, many times because stains encompass a strong color contrast, whereas holes are just a discontinuity in the fiber pattern, with the color contrast coming only from shadowing. Although the YOLOv5l6 model had a higher AP value (0.747), the YOLOv5m6 model exhibited a higher recall value (0.633). On the other hand, the larger model had a higher precision value (0.915). The primary reason for the higher AP value of the YOLOv5l6 model is the significant difference in precision compared to the YOLOv5m6 model. However, considering the context of this application, prioritizing recall over precision may be more beneficial. In other words, it is preferable for the model to have fewer false negatives than false positives. Figure 7 illustrates an example of a false positive, where the buttonholes were misinterpreted as a defect. This highlights the importance of having representative images that include such scenarios.

**Figure 8:**
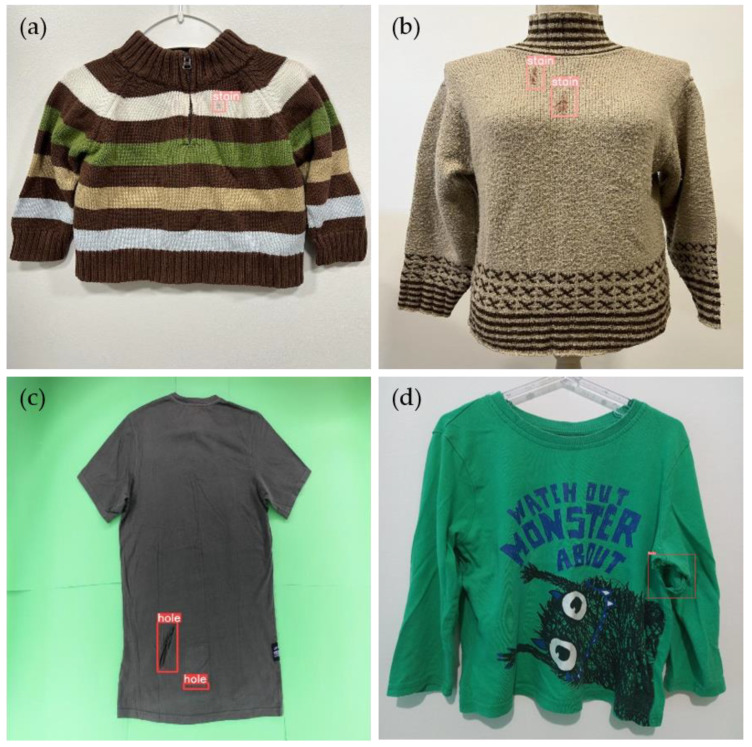
Examples of predicted images from the YOLOv5l6: (**a**) single stain detection, (**b**) multiple stain detection, (**c**) multiple hole detection, and (**d**) hole detection near the seam.

Based on the performance of the models across all experiments, the model YOLOv5l6 exhibited the best generalization to unseen data, i.e., the test dataset, when compared to the other models. Figure 8 displays predicted images that encompass various scenarios, including variations in illumination, backgrounds, multiple defects, as well as challenging areas.

The main constraint of using this model in a practical context is the computational cost. Such impact was evaluated through the calculation of the inference time for the test with the dataset. Table 7 exhibits the results of the inference time on the test dataset. 

These results suggest that, despite incurring computational costs, all models are believed acceptable due to the negligible required time. Thus, the findings indicate that implementing object detection technology with augmented data may be a successful strategy for identifying defects in clothing. This study stands out from previous research work as it evaluates defect detection on clothing overall, instead of focusing on zoomed-out images of defects on stretched textiles and without a background. Upon comparing our dataset with those from textile fabrics, it became clear how challenging the task presented in this work is, mainly due to the presence of certain features such as buttonholes, which could potentially be interpreted as defects. Furthermore, this approach was proven to be effective in highly demanding contexts, namely with wrinkled textiles, various backgrounds, different illumination, and diverse patterns.

## 5. Conclusions and Future Work

Blind individuals face daily challenges with simple tasks, namely related to clothing and style, which are critical components of one’s personal identity. Assistance from family or friends is often required to support daily dressing-up tasks and, many times, such help is essential for detecting defects on clothes that, otherwise, would go unnoticed. Therefore, defect detection in clothing is of the utmost importance for blind individuals to feel comfortable and confident with their appearance. With this premise in mind, the present study aimed at improving a defect detection system for clothing, following a three-step methodology based on: (i) enlarging the dataset, (ii) introducing data augmentation, and (iii) introducing defect classification.

The detection and classification of clothing defects was successfully carried out with a deep learning approach. An enhanced dataset was constructed with new types of stains and holes. Through the fine-tuning of three models from the YOLOv5 object detector, a total of three experiments were carried out. Data augmentation was demonstrated to be essential for a better generalization of the model, allowing to achieve higher precision results. However, the recall values demonstrated that the model can still be improved to minimize false negatives. Maximum precision and recall values of 0.76 and 0.747 were achieved, respectively, with the model YOLOv5l6 for defect detection and defect classification. The detection of holes was found to be more challenging than the detection of stains, which emphasizes the importance of integrating the findings of this study in an automatic wardrobe that could take multiple images from the perspective of different clothing items.

The dataset built in this work demonstrated that object detection technology can be used to accurately and autonomously detect and classify defects on clothing. Moreover, it represents the first step for the creation of a mobile application that can effectively detect multiple defects on clothing, based on the integration of these findings in an automated closet system as a future step. Overall, the main objective of this study was accomplished, since a system that enables blind people to automatically identify clothing and detect multiple defects in garments was successfully developed and tested, thereby providing them with greater independence and autonomy, while contributing to an improved quality of daily life. 

## Figures and Tables

**Table 1:** Literature overview on textile fabric defect detection, including the author, year, method, dataset, defect classes, and metrics.

Author	Year	Method	Dataset	Defect Classes	Metrics
Hang et al. [25]	2018	DL object detection (YOLOv2)	Collected dataset: 276 manually labeled defect images	3	IoU: 0.667
Mei et al. [26]	2018	Multiscale convolutional denoising autoencoder network model	Fabrics dataset: ca. 2000 samples of garments and fabrics	-	Accuracy: 83.8%
			KTH-TIPS	-	Accuracy: 85.2%
			Kylberg Texture: database of 28 texture classes	-	Accuracy: 80.3%
			Collected dataset: ms-Texture	-	Accuracy: 84.0%
He et al. [27]	2020	DenseNet-SSD	Collected dataset: 2072 images	6	mAP: 78.6%
Jing et al. [28]	2020	DL segmentation (Mobile-Unet)	Yarn-dyed Fabric Images (YFI): 1340 images composed in a PRC textile factory.	4	IoU: 0.92; F1: 0.95
			Fabric Images (FI): 106 images provided by the Industrial Automation Research Laboratory of the Department of Electrical and Electronic Engineering at Hong Kong University	6	IoU: 0.70; F1: 0.82
Han et al. [29]	2020	Stacked convolutional autoencoders	Synthetic and collected dataset	-	F1: 0.763
Mohammed et al. [30]	2020	A multilayer perceptron with a LM algorithm	Collected dataset: 217 images	11	Accuracy: 97.85%
Xie et al. [31]	2020	Improved RefineDet	TILDA dataset: 3200 images; only 4 classes were used from 8 in total, resulting in 1597 defect images.	4 of 8	mAP: 80.2%; F1: 82.1%
			Hong Kong patterned textures database: 82 defective images.	6	mAP: 87.0%; F1: 81.8%
			DAGM2007 Dataset: 2100 images	10	mAP: 96.9%; F1: 97.8%
Huang et al. [32]	2021	Segmentation network	Dark redfFabric (DRF)	4	IoU: 0.784
			Patterned texture fabric (PTF)	6	IoU: 0.695
			Light blue fabric (LBF)	4	IoU: 0.616
			Fiberglass fabric (FF)	5	IoU: 0.592
Kahraman et al. [33]	2022	Capsule Networks	TILDA dataset	7	Accuracy: 98.7%

**Table 2:** Defect class distribution, focusing on the two main class defects of interest for the present work.

Class	Number of Defects
Stain	323
Hole	324

**Table 3:** Hyper-parameters (image size, optimizer, learning rate, and batch size) of model experiments.

Parameters	Value
Image Size	1024 × 1024 pixels
Optimizer	Stochastic gradient descent (SGD)
Learning Rate	0.01
Batch Size	16

**Table 4:** Main results from the fine-tuning of the models without data augmentation for defect detection (precision, recall, and AP at IoU = 0.50).

Model	Precision	Recall	AP at IoU = 0.50
YOLOv5s6	0.85	0.41	0.62
YOLOv5m6	0.83	0.53	0.66
YOLOv5l6	0.86	0.60	0.73

**Table 5:** Main performance results of the models after introducing data augmentation for defect detection (precision, recall, and AP at IoU = 0.50).

Model	Precision	Recall	AP at IoU = 0.50
YOLOv5s6	0.78	0.54	0.69
YOLOv5m6	0.80	0.63	0.74
YOLOv5l6	0.94	0.58	0.76

**Table 6:** Performance results of each model with data augmentation and defect classification (precision, recall, and AP at IoU = 0.50).

Model	Class	Precision	Recall	AP at IoU = 0.50
YOLOv5s6	all	0.849	0.538	0.688
	hole	0.836	0.448	0.610
	stain	0.863	0.628	0.765
YOLOv5m6	all	0.823	0.593	0.726
	hole	0.696	0.552	0.656
	stain	0.950	0.633	0.796
YOLOv5l6	all	0.915	0.543	0.747
	hole	0.889	0.552	0.741
	stain	0.941	0.533	0.753

**Table 7:** Inference time on the test dataset for the different YOLOv5 models tested.

Model	Inference Time (s)
YOLOv5s6	0.0092
YOLOv5m6	0.0112
YOLOv5l6	0.0157
